# Psychological Components of Disease Stigma Across Illnesses: Associations with Cultural and Personal Factors

**DOI:** 10.3390/bs16020295

**Published:** 2026-02-19

**Authors:** Shiming Yao, Jiajia Zhu, Yan Mu

**Affiliations:** 1 State Key Laboratory of Cognitive Science and Mental Health, Institute of Psychology, Chinese Academy of Sciences, Beijing 100101, China; yaosm@psych.ac.cn (S.Y.); zhujj@psych.ac.cn (J.Z.); 2 Department of Psychology, University of Chinese Academy of Sciences, Beijing 101408, China

**Keywords:** disease stigma, cultural tightness, self-control, self-esteem

## Abstract

Understanding public stigma against patients (also known as disease stigma)—negative attitudes or discriminatory responses toward individuals with a disease—is essential for improving health outcomes and fostering inclusive communities. In this study, 279 participants rated their responses toward eight disease groups (e.g., HIV/AIDS, COVID-19, and depression). Using multiple factor analysis, we identified three components of disease stigma: exclusionary (e.g., avoidance and harmful evaluation), prosocial (e.g., sympathy and helping), and attribution (blame/responsibility). Confirmatory factor analysis supported this three-component structure. Repeated-measures ANOVAs revealed systematic differences across diseases: COVID-19 and schizophrenia elicited stronger exclusionary responses, depression evoked the strongest prosocial responses, and HIV/AIDS was associated with the highest attribution of blame. Linear mixed-effects models further indicated that perceived cultural tightness was positively associated with the attribution component, self-control was positively associated with the prosocial component, and higher self-esteem was linked to greater exclusionary responses. Furthermore, network analysis showed dense within-component clustering (e.g., trust—negative evaluation; sympathy—helping) and a peripheral positioning of attribution within the stigma network. These findings provide insights into the psychological components of disease stigma and its cultural and personal correlates, providing targets for component-specific stigma reduction strategies.

## 1. Introduction

### 1.1. Conceptual Framework for Disease Stigma

Public stigma against patients—negative attitudes and discriminatory responses toward individuals with a disease—is a central form of disease stigma. Disease stigma is not reducible to a single negative attitude; rather, it is a multidimensional social process in which certain individuals or groups are labeled and stereotyped, separated from others, and subjected to status loss and discrimination within asymmetrical relations of social power (Link & Phelan, 2001). Importantly, stigma can operate through multiple channels (e.g., affective reactions, cognitive appraisals, and behavioral tendencies) and may take ambivalent forms rather than being uniformly hostile. For instance, reactions such as sympathy or willingness to help can coexist with avoidance, fear, or blame and remain stigma-relevant when they reflect differential, identity-marking, or paternalistic responses that reinforce social boundaries rather than genuine social inclusion (Corrigan et al., 2003). 

Public stigma toward people with a health condition can have substantial consequences, including reduced opportunities, social exclusion, and barriers to help-seeking, and it can be internalized as self-stigma, undermining psychological well-being and recovery (Major et al., 2018; Vogel & Wade, 2022). Those suffering from disease stigma often internalize the negative attitudes of society, leading to lower self-esteem (Link et al., 2001; Maharjan & Panthee, 2019), inducing shame (Chapple et al., 2004; Garthwaite, 2016; Waller et al., 2007), and deterring treatment-seeking (Fischer et al., 2019). Moreover, it also marginalizes patients (Li et al., 2020; Ramaci et al., 2020), erodes their social identity (Goffman, 2009), and weakens societal cohesion, especially during health crises (Mitchell et al., 2021). For instance, during COVID-19, disease stigma extended to associated groups—including healthcare workers (Grover et al., 2020), family (Chew et al., 2021), and even mask-wearers (O’Brien et al., 2024)—resulting in discrimination and reduced care quality (Hatzenbuehler et al., 2013). Therefore, there is an urgent need to understand its psychological underpinnings and influencing factors to guide policy interventions.

### 1.2. Psychological Components of Disease Stigma

Notably, prosocial reactions to disease stigma are not inherently stigma-free. When supportive intentions are selectively directed toward a labeled group (relative to healthy individuals), they may reflect norm-guided, paternalistic, or role-based benevolence rather than unconditional social inclusion and therefore remain stigma-relevant as a form of unequal, identity-marking social treatment. In the present study, we argue that disease stigma is a multidimensional concept, including three interrelated components: (a) exclusionary reactions (e.g., avoidance, fear, and devaluations), (b) prosocial or affirming reactions (e.g., sympathy, willingness to help, supportive intentions) that may be contingent, asymmetric, or paternalistic, and (c) attributional appraisals (e.g., perceived responsibility or blame) that shape moral and social evaluations of the target. This framework aligns with prior accounts showing that pity or sympathy can motivate helping while simultaneously reflecting a stigmatized construal of the target (Batson et al., 2002; Corrigan et al., 2003) as well as with work suggesting that pity-based helping often embodies paternalistic social evaluations (Fiske, 2002).

Previous evidence on disease stigma remains inconsistent, largely due to different conceptualizations and measurements across illness types. Mental illness stigma has been operationalized through constructs such as perceived dangerousness (Pescosolido et al., 2013), internalized shame (Ahmedani, 2011), or social isolation (Earnshaw et al., 2013). In contrast, pandemic-related stigma focuses on fear-driven avoidance and moral blame (T. M. Zhang et al., 2021; Zhu et al., 2022). Although these stigmas may share fear as a common emotional core, it remains unclear whether they reflect distinct components or merely variations in a shared structure. This highlights a key issue: the field lacks an integrated framework that captures both the shared and unique psychological components of disease stigma across health conditions.

### 1.3. Core Psychological Processes in Disease Stigma

Disease stigma can elicit two divergent social responses ranging from prosocial concern to social exclusion—depending on how the target is construed. A central process underlying these divergent responses is perceived controllability, which refers to the extent to which a condition is viewed in lay judgments as preventable or behavior-linked and therefore under personal control. Conditions commonly attributed to individual behavior (e.g., substance use disorders, obesity, or smoking-related illnesses) are typically perceived as more controllable, whereas conditions attributed to external, genetic, or random causes (e.g., cancer or congenital disorders) are generally perceived as less controllable. Accordingly, targets associated with low perceived controllability conditions are more likely to be construed as victims and to elicit sympathy and support (Dovidio et al., 2017b; Rusch et al., 2010). However, when they are perceived as responsible for their illness or as norm violators (Lauber, 2008), stigma more often manifests as distrust (M. Zhang et al., 2020) and limited support (Wallhed Finn et al., 2023; Weiner et al., 1988). These divergent reactions are further shaped by sociocultural and moral evaluations: dehumanization and moral threat accounts suggest that stigmatized individuals may be viewed as socially deviant or morally compromised (Boysen et al., 2023; Haslam, 2006), prompting shame and distancing (Rozin et al., 2006; Weiner, 1995). As a result, social reactions are often ambivalent—people may feel both sympathy and suspicion toward targets seen as simultaneously vulnerable and blameworthy (Batson et al., 2002; Corrigan et al., 2003). This ambivalence is well explained by the approach–avoidance framework (Chen & Bargh, 1997; Davidson et al., 1990; Neumann & Strack, 2000), which posits that stigma can elicit simultaneous urges to help and to withdraw (Elliot et al., 2006).

Existing literature suggests that fear is a central driver of stigma across both mental (Brockington et al., 1993; Lundberg et al., 2007; Taylor & Dear, 1981) and physical illness (Person et al., 2004; Wasim et al., 2023). Fear often triggers avoidance, exclusion, and negative attributions—viewing patients as irresponsible (Corrigan & Miller, 2004; Jones, 1984) or undeserving (Corrigan et al., 2002; Socall & Holtgraves, 1992; Weiner et al., 1988). These responses align with the fear-based exclusion model, rooted in behavioral immune system theory, which suggests humans evolved automatic emotional reactions to potential sources of contagion (Phelan et al., 2008; Schaller & Park, 2011). For instance, individuals with infectious diseases like COVID-19 (Zhu et al., 2022) or HIV/AIDS (Herek, 1999) often face fear-driven distancing and social exclusion.

Stigma involves both automatic affective responses and higher-order cognitive regulation. For example, attitudes toward people with HIV/AIDS, initially fear-based, improve after a delay, reflecting cognitive adjustment (Pryor et al., 1999). Neuroimaging shows that prefrontal control processes override negative reactions to stigmatized individuals (Krendl et al., 2012). The higher-order cognitive component of stigma is supported by the dual-process model (Reeder & Pryor, 2008), which distinguishes emotion-driven associations (e.g., linking illness with danger) (Fazio & Olson, 2003) from deliberate, rule-based judgments shaped by norms and beliefs (Best & Arseniev-Koehler, 2023; Gawronski & Bodenhausen, 2006). Attributional beliefs also shape stigma’s intensity and moral tone (Corrigan et al., 2003; Weiner et al., 1988)—controllable illnesses tend to elicit blame, whereas uncontrollable conditions evoke sympathy and support (Corrigan, 2000; Johnson-Kwochka et al., 2024; Weiner et al., 1988).

### 1.4. Cultural Factor and Disease Stigma

As a key contextual factor, culture shapes not only what is stigmatized but also how stigma is expressed by structuring normative expectations and the acceptable range of deviation (Misra et al., 2021; Zay Hta et al., 2024). One influential framework is cultural tightness-looseness (TL), which captures the strength of social norms and the tolerance for deviance. Tight cultures are characterized by stronger norms and stricter sanctioning of norm violators, whereas loose cultures show weaker norms and greater permissiveness toward deviance (Gelfand et al., 2011; Harrington & Gelfand, 2014). Substantial within-nation variation in norm strength has also been documented, with a validated tightness-looseness index and rankings across the 50 U.S. states (Harrington & Gelfand, 2014). Beyond documenting cross-cultural variation in norm strength, tightness-looseness has also been linked to evaluative bias: tighter nations and U.S. states show higher explicit prejudice and stronger implicit bias in large-scale Project Implicit IAT data (Jackson et al., 2019). This normative ecology is directly relevant to disease stigma because illness can be constructed as a form of “deviation” that threatens collective functioning, cleanliness, or social order, thereby eliciting moralized evaluations and regulatory social responses.

We propose that perceived tightness is associated with heightened disease stigma through two complementary cultural pathways. First, stronger norm enforcement in tighter contexts may increase the tendency to construe illness as a controllable, responsibility-relevant outcome or as a norm violation, thereby strengthening punitive attributions and exclusionary reactions (Jones, 1984; Mason & Morris, 2010; Weiner et al., 1988; Zaw, 2006). Second, tighter contexts may also heighten role-based obligations and normative duties to “help” or “manage” vulnerable targets (e.g., stronger family/community obligations), which can elevate supportive intentions in ways that remain stigma-relevant, because such help may be conditional, paternalistic, or identity-marking rather than unequivocal acceptance (Corrigan et al., 2003; Fiske, 2002; Gelfand et al., 2011). Accordingly, cultural tightness–looseness is expected to be associated with both heightened exclusionary responses and elevated, yet potentially paternalistic, prosocial reactions toward stigmatized illness targets, contributing to the ambivalent patterning of disease stigma. We therefore included perceived cultural tightness as a key contextual factor in predicting the stigma components.

### 1.5. Individual Factors and Disease Stigma

Personal factors, such as self-control and self-esteem, may play important roles in shaping how individuals perceive and respond to stigmatized groups. Stigma is a psychologically disruptive process that disrupts interpersonal relationships (Frost, 2011; Major & Eccleston, 2004; Rossler, 2016; Sayce, 1998). Studies suggest that prejudice regulation—such as in attitudes toward HIV patients—depends increasingly on self-regulatory processes like self-control (Pryor et al., 2004; Reeder & Pryor, 2008). Self-control, as a core executive function, helps regulate emotional reactions during interpersonal interactions (Diamond, 2013). This capacity not only suppresses immediate stigmatizing reactions but also facilitates the internalization of social norms that discourage prejudice (Conner et al., 2023). Thus, self-control may be linked to social and cognitive components of disease stigma.

Self-esteem may also influence stigmatizing attitudes, though findings are mixed. While low self-esteem is associated with devaluing others as a compensatory strategy (Abrams & Hogg, 1988; Fiske et al., 2018), individuals with higher self-esteem tend to exhibit stronger identification with their own social groups, which can sometimes exacerbate prejudiced attitudes towards out-groups (Baumeister et al., 2003; Crocker & Major, 1989; Jetten et al., 2015). Given these opposing trends, this study explores whether the two self-related variables—self-control and self-esteem—play similar or distinct roles in their associations with disease stigma.

### 1.6. Research Gaps

Despite substantial progress, research on disease stigma remains fragmented in both concepts and measures, making it difficult to compare findings across conditions and to synthesize conclusions. First, stigma has been defined and operationalized differently across studies, making it difficult to compare findings or synthesize conclusions. Second, stigma-related measures (e.g., fear, blame, and social distance) vary widely across studies, making comparison and synthesis difficult. Third, many studies examine stigma associated with certain diseases in isolation (e.g., mental illness, HIV, and COVID-19) without identifying shared mechanisms (Pryor & Reeder, 2011). Fourthly, existing theories of stigma highlight diverse mechanisms at different levels of explanation, ranging from evolutionary disease-avoidance defenses (e.g., the behavioral immune system theory) (Kurzban & Leary, 2001) to attributional processes emphasizing responsibility and controllability judgments (e.g., the attribution theory) (Corrigan et al., 2003; Weiner, 1995), social sequences describing the translation of stereotypes into prejudice and discriminatory actions (Link & Phelan, 2001; Major et al., 2018), cultural models emphasizing the role of norms and tightness–looseness (Pescosolido et al., 2013; Yang et al., 2007), as well as structural taxonomies of stigmatized conditions (Pachankis et al., 2018). However, these frameworks often emphasize either upstream features of stigmatized targets or condition-specific dynamics. It is still unclear whether commonly used stigma indicators reflect underlying generalizable components, how these components differ across disease contexts, and how they are influenced by cultural and personal factors.

As a result, it remains unclear (a) whether commonly used stigma indicators reflect a small set of generalizable psychological components across diseases, (b) how these components vary by condition, and (c) how cultural and individual differences systematically predict these components.

### 1.7. The Current Study

The present study aimed to identify core psychological components of disease stigma across a diverse set of health conditions and to test how cultural context and individual differences shape these components. Using multiple infectious (e.g., COVID-19, flu, and Ebola) and mental conditions (e.g., depression and schizophrenia), and drawing on previous research (Corrigan et al., 2003; Pescosolido et al., 2008; Yang et al., 2007).

First, we hypothesized that disease stigma is expected to exhibit a multidimensional structure, rather than reflecting a single homogeneous construct. Different stigma indicators are hypothesized to cluster into distinct but interrelated components that capture qualitatively different psychological responses toward stigmatized groups (e.g., exclusionary, prosocial, and attribution-related tendencies). This structure could be empirically examined using factor-analysis approaches (e.g., multiple factor analysis, MFA). Second, we further expected that the identified stigma components would vary systematically across different disease target groups, reflecting meaningful differences in how diseases and conditions are socially evaluated. We aimed to examine target-group differences using repeated-measures ANOVAs with Bonferroni-adjusted contrasts. Third, we predicted that cultural and personal factors would show different associations with distinct components of disease stigma, rather than exerting uniform effects. Particularly, individual perceived cultural tightness is hypothesized to be associated with norm-relevant evaluative components of disease stigma, with stronger associations expected for components that involve judgments of responsibility and social regulation. We expected that self-control would be associated with stigma components reflecting regulated and affiliative responses. Given mixed prior evidence, self-esteem was included and examined as an exploratory predictor of stigma components. We tested the associations between the predictors and stigma components using linear mixed-effects models. Finally, we conducted a psychological network analysis to visualize and investigate how different stigma components and indicators relate to one another, expecting relatively stronger within-component connectivity than cross-component connectivity at the descriptive level.

By modeling disease stigma as a set of separate components and estimating these components across multiple illness targets relative to a healthy baseline, the present study contributes to stigma research in two ways. First, it provides an empirically grounded, cross-condition component structure—exclusionary reactions, prosocial/affirming reactions, and attributional appraisals—that helps reconcile the wide ranges of measures and mixed operationalizations in prior work by clarifying which aspects of stigma linger together and how they vary across diseases.

Second, the study identifies context- and personal-level correlates of these stigma components. Specifically, we examine perceived cultural tightness–looseness as an indicator of cultural context and self-related individual differences (self-control and self-esteem) as potential predictors of baseline-corrected component scores. Practically, distinguishing component-specific predictors can inform more targeted stigma-reduction strategies—for example, interventions can be tailored to reduce exclusionary responses and punitive attributions in contexts where norm enforcement is stronger, while also recognizing that seemingly supportive reactions (e.g., “helping” or suppressing negative emotions) may reflect differential, potentially identity-marking treatment rather than unequivocal acceptance.

## 2. Materials and Methods

### 2.1. Participants

Participants were recruited via Wenjuanwang (https://www.wenjuan.com), an online survey platform widely used in Mainland China. This study reanalyzed data from a larger project on social norms and well-being, with a specific focus on disease stigma. A priori power analysis using G*Power 3.1 (Faul et al., 2009) indicated that a minimum of 23 participants was sufficient for a repeated-measures ANOVA (*f* = 0.25, *α* = 0.05, 1 − *β* = 0.95). However, to ensure sufficient power for multiple factor analysis, regression, and network modeling, a larger sample of at least 250 was targeted (Epskamp et al., 2018; Goretzko et al., 2021). Of 348 initial respondents, 69 were excluded for incomplete responses or failed attention checks, yielding a final analytic sample of 279 participants (*M_age_* = 24.71, *SD* = 7.31; 177 self-identified as females, 102 self-identified as males), with a validity response rate of 80.17%. All participants provided informed consent and received monetary compensation (CNY 20).

### 2.2. Measures

Participants completed a set of self-reported questionnaires assessing attitudes and emotional responses toward a range of disease groups, together with cultural and personal characteristics. To capture disease stigma as a multifaceted pattern of differential social treatment, we selected ten stigma indicators representing three theoretically motivated domains: (i) exclusionary/avoidance-related responses (fear, avoidance, harmful evaluation, and negative evaluation), (ii) prosocial/affiliative reactions (sympathy, helping, suppression of negative emotion, and trust), and (iii) attributional and norm-relevance appraisals (attribution/blame and social deviance). These indicators were chosen because they are frequently used across major stigma frameworks (e.g., behavioral immune-system accounts emphasizing fear/avoidance; attribution models emphasizing blame/controllability; and sociocultural accounts emphasizing deviance and moralized evaluation), allowing direct cross-condition comparison within a single measurement set (Kurzban & Leary, 2001; Pachankis et al., 2018; Stangl et al., 2019). Each item was rated on a 7-point Likert scale (1 = very unlikely, 7 = very likely), indicating how likely participants would be to experience each feeling or reaction toward a target individual (e.g., “I would feel fearful if interacting with someone infected by this disease”).

For each target group, participants first read a block header specifying the target identity (e.g., “a person with influenza,” “a person with COVID-19,” “a person with depression,” “a person with schizophrenia,” or “a healthy person”). They then rated the same fixed act of reaction items using an identical instruction/stem (e.g., “When encountering this person, how likely would you …”, see Table S1 for complete items). Thus, item wording remained constant across conditions, with only the target label in the block header varying between groups.

Participants rated the same 10 items for eight target groups: COVID-19, flu, SARS, HIV/AIDS, Ebola, depression, schizophrenia, and a healthy control. Our objective was not to exhaustively sample all stigmatized health conditions, but to achieve broad theoretical coverage within a feasible within-subject design. Accordingly, disease targets were selected to (a) span infectious vs. non-infectious conditions, (b) vary in perceived contagion and lethality (e.g., flu vs. Ebola), and (c) represent distinct, well-established stigma-relevant appraisal profiles documented in prior research (e.g., moralized responsibility and blame in HIV/AIDS versus unpredictability and social distance concerns in schizophrenia). A healthy control target was included to provide an individual-level interpersonal baseline; component scores were therefore interpretable as baseline-corrected deviations (disease minus healthy) rather than absolute endorsement levels. Please see Supplementary Materials S1 for details of disease target selection.

To examine contextual influences, participants also reported their perceived cultural tightness-looseness using the 6-item General Cultural Tightness-Looseness Scale (*α* = 0.64; e.g., “In the province and city that you live, there are many social norms that people are expected to follow”) (Gelfand et al., 2006). Self-control was assessed with a 13-item self-control scale (Tangney et al., 2004) (*α* = 0.80; e.g., “I am able to work effectively toward long-term goals”). Self-esteem was measured with the 10-item Rosenberg Self-Esteem Scale (Rosenberg, 1965) (*α* = 0.81; e.g., “I am satisfied with myself”), with half of the items reverse-coded. All of these scales used a 7-point Likert response format (1 = strongly disagree, 7 = strongly agree).

We include demographic covariates—age, gender, education, and SES—because prior research on stigma attitudes (Dessie & Zewotir, 2024; Fox et al., 2018; Phelan, 2005), as well as studies on health-related responses and perceived risks (Jane Ling et al., 2023; Kang et al., 2025), show that these factors can systematically covary with stigma-relevant responses, such as disease-related risk perception, moralized blame, and negative social evaluation. SES was measured by a 10-rung visual ladder scale (Adler et al., 1994). These demographic variables were included as covariates in the linear mixed-effects models.

### 2.3. Analysis Approach

Stigma ratings (10 items) across eight target groups were z-standardized and submitted to multiple factor analysis (MFA) (Pagès, 2002) using the *FactoMineR* package in R (Lê et al., 2008; available at the Comprehensive R Archive Network, CRAN: FactoMineR package page; more details in Data Availability section). MFA was chosen because it performs a principal-component decomposition within each variable block (here, disease groups) before integrating those blocks through a weighted global analysis, thereby preserving block-specific characteristics while revealing their common structure. The number of components was determined based on scree plots and explained variance (EV) (Kaiser, 1960). The resulting three-component solution was further validated via confirmatory factor analysis (CFA) using the *lavaan* package (Rosseel, 2012), with parameters estimated via robust maximum likelihood (MLR) to account for potential non-normality. Model fit was evaluated using robust *χ*^2^ tests, Comparative Fit Index (CFI), Tucker–Lewis Index (TLI), Root Mean Square Error of Approximation (RMSEA), and Standardized Root Mean Square Residual (SRMR).

To test group differences, repeated-measures ANOVAs were conducted for each component, with target group (eight levels: COVID-19, flu, SARS, HIV/AIDS, Ebola, depression, schizophrenia, and healthy control) as the within-subject factor. Greenhouse-Geisser correction was applied if Mauchly’s test indicated violations of sphericity, and Bonferroni-adjusted two-tailed post hoc comparisons were performed on pairwise contrasts.

To examine how cultural and individual differences predict stigma components across diseases, linear mixed-effects models were estimated with the *lme4* (Bates et al., 2015) and *lmerTest* (Kuznetsova et al., 2017) packages. To isolate these effects from general endorsement tendencies, component scores for each disease were recalculated after subtracting the corresponding healthy control scores, and the adjusted scores served as dependent variables. This baseline correction reduces between-person differences in general response tendencies and focuses inference on disease-specific deviations from participants’ own interpersonal baseline. Perceived cultural tightness-looseness, self-control, and self-esteem were entered as fixed effects, with disease type and participants’ ID modeled as random intercepts. Demographic covariates (age, gender, education, and subjective SES) were also included as fixed effects. When models failed to converge due to a singular fit, the offending random slopes were removed iteratively until convergence.

Finally, we conducted a psychological network analysis to visualize associations among stigma items using the Extended Bayesian Information Criterion (EBIC)–glasso (*γ* = 0.5) implemented in the *mgm* (Haslbeck & Waldorp, 2015) and *bootnet* (Epskamp et al., 2018) packages. Disease lethality (case-fatality rate, CFR; ordered across diseases) was entered as a continuous covariate to partial out the influence of objective threat. Centrality indices (strength, betweenness, closeness, and expected influence) were computed to assess the relative importance of each stigma item. Edge-weight accuracy and centrality stability were evaluated through 5000 non-parametric bootstrap iterations.

## 3. Results

### 3.1. The Psychological Components of Disease Stigma

**Figure 1:**
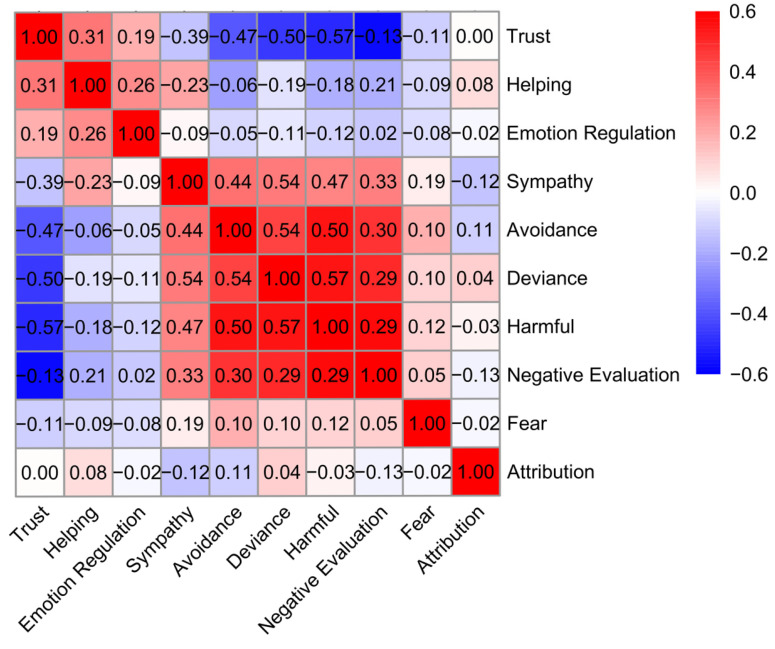
Correlation results. Heat map of intercorrelations among ten stigma indicators across target groups. Warmer colors denote positive and cooler colors negative associations. Correlations close to zero (*r* = [−0.05, 0.05]) are non-significant.

We examined correlations among ten indicators of stigma—including fear, avoidance, harmful evaluation (perceived harmfulness to society), sympathy, helping (willingness to help), emotion regulation (willingness to regulate potential negative affects towards target groups), trust (willingness to trust), attribution (responsibility/blame), social deviance (perceived deviation from social norms), and negative evaluation (general disapproval; see Materials and Methods section). Correlation analysis revealed that most indicators were significantly associated, though the strength of these associations varied (Figure 1). For instance, avoidance correlated positively with harmful evaluation and negative evaluation and negatively with trust and helping. Notably, no significant association was found between avoidance and attribution. These varied associations highlight conceptual differences among stigma indicators and underscore the need to examine the underlying latent structure of stigma.

**Figure 2:**
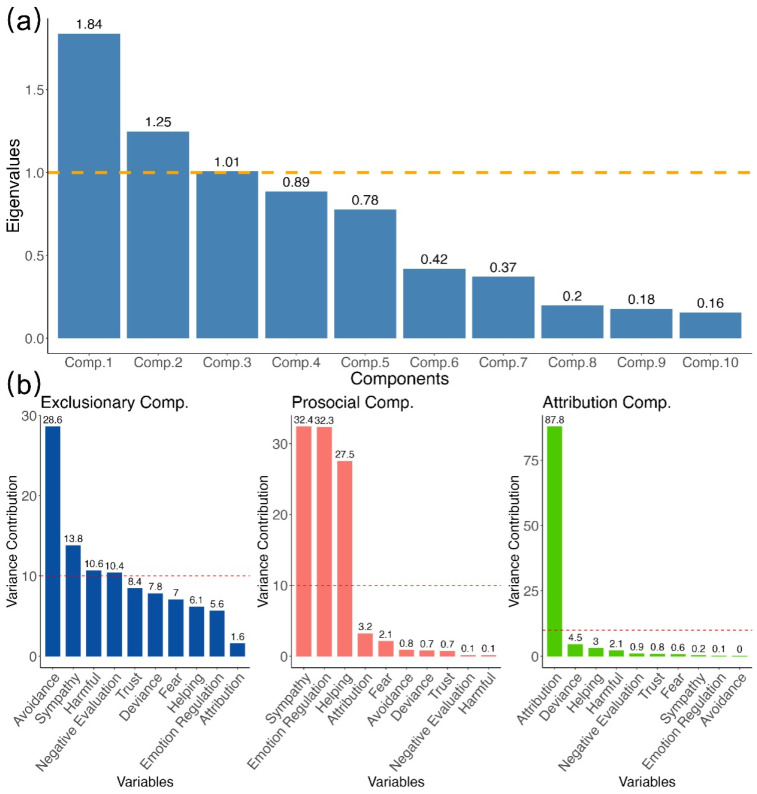
Multiple factor analysis. Scree plot and item-loading bars reveal the three stigma components—exclusionary, prosocial, and attribution—together accounting for 58% of the variance. (**a**) Scree plot showing the eigenvalues of components (comp.1–comp.10). The horizontal dashed line at eigenvalue = 1.0 marks the Kaiser criterion; components above this line are typically considered to explain more variance than an averaged single variable. (**b**) Variance contribution (in %) for the MFA components. The horizontal dashed line at 10% indicates the expected equal contribution when 10 items contribute uniformly (i.e., 1/10 of the total contribution); items above this line contribute more than the equal-share benchmark.

To extract the underlying components of disease stigma indicators, we conducted a multiple factor analysis (MFA). The analysis revealed a three-component structure accounting for 57.84% of the variance (Figure 2a, Supplementary Table S1). The first component, labeled exclusionary, reflected negative reactions such as avoidance, harmful evaluation, and (dis)trust (Eigenvalue = 1.84, EV = 25.97%). The second, prosocial, encompassed supportive responses including sympathy and helping (eigenvalue = 1.25, EV = 17.64%). The third, attribution, captured judgments of personal responsibility and blame (eigenvalue = 1.01, EV = 14.23%).

A confirmatory factor analysis (CFA) was then conducted to validate this three-factor structure. The model showed marginal fit (scaled *χ*^2^(32) = 541.19, *p* < 0.001, Robust CFI = 0.896, Robust TLI = 0.853, Robust RMSEA = 0.088, 90% CI [0.081, 0.095], SRMR = 0.051; see explained variances of each variable in Figure 2b). While CFI and TLI were slightly below the conventional 0.9 cutoff, SRMR indicated acceptable residual fit, and the factor loadings aligned with the MFA-derived structure, a recommended practice when validating exploratory solutions with confirmatory models (Abdi et al., 2013; Brown, 2015; Marsh et al., 2014).

### 3.2. Differences in Stigma Components Among Disease Groups

We conducted repeated-measures ANOVAs with target group (eight levels: COVID-19, flu, SARS, HIV/AIDS, Ebola, depression, schizophrenia, and a healthy control) as within-subject variable and found significant main effects of target group on all three components: exclusionary, *F*(4.51, 1199.03) = 454.32, *p* < 0.001, partial *η*^2^ = 0.631; prosocial, *F*(3.92, 1042.27) = 57.65, *p* < 0.001, partial *η*^2^ = 0.178; and attribution, *F*(5.12, 1361.58) = 34.94, *p* < 0.001, partial *η*^2^ = 0.116; Supplementary Tables S2 and S3). These findings suggest that stigma components vary systematically across different target groups, thereby supporting the validity of the three-component structure.

**Figure 3:**
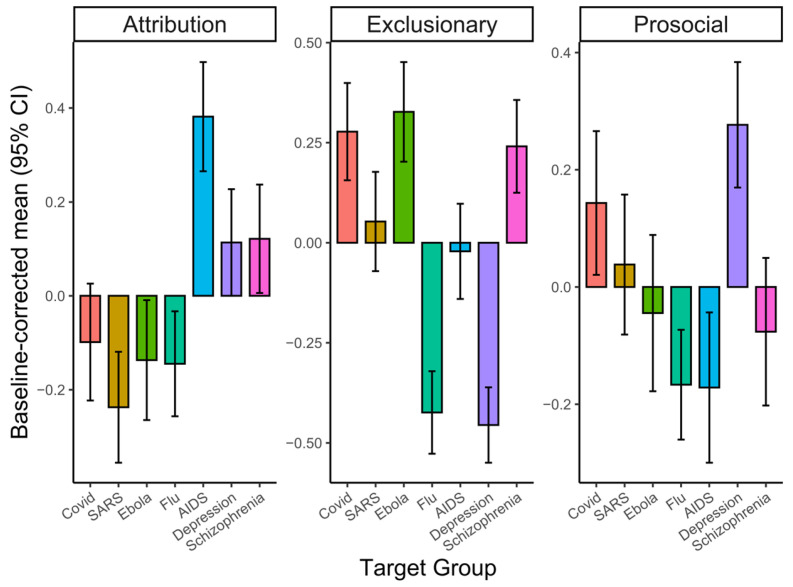
ANOVA results. Baseline-corrected mean scores for the three stigma components are shown by target groups (error bars = 95% CI).

Bonferroni-adjusted pairwise comparisons showed that disease groups scored higher than the healthy control on the exclusionary and prosocial components (all *p*s < 0.001; Figure 3). Specifically, for the exclusionary component, COVID-19, Ebola, and schizophrenia yielded the highest scores, while flu and depression scored the lowest. For the prosocial component, depression showed the highest responses, and COVID-19 also scored higher than several other conditions (e.g., flu, HIV/AIDS, schizophrenia), though not SARS. For the attribution component, HIV/AIDS scored significantly higher than all other groups (all *p*s < 0.001), whereas depression, schizophrenia, and COVID-19 did not differ significantly from the healthy control. Full statistics are provided in Supplementary Tables S2–S4.

### 3.3. Associations of Cultural and Personal Factors with Stigma Components

**Figure 4:**
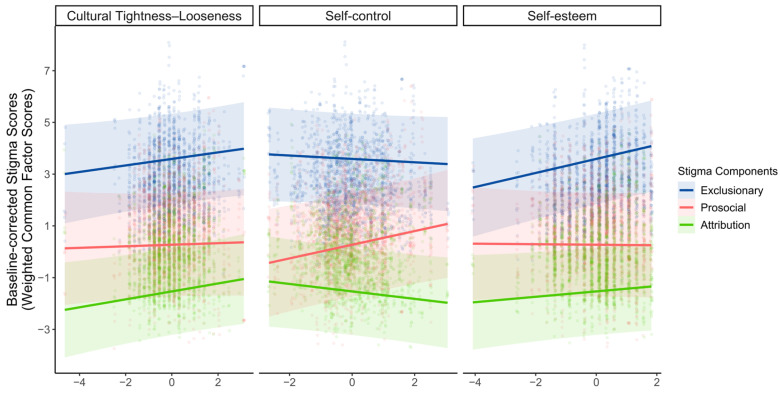
Linear mixed-effects results. The associations between perceived cultural tightness–looseness, self-control, self-esteem (panels arranged top to bottom), and baseline-corrected component scores averaged across targets; ribbons indicate 95% confidence bands. Colors denote stigma components (Exclusionary = blue, Prosocial = red, Attribution = green). Lines show model-implied marginal predictions, points represent partial-residual observations, and ribbons indicate 95% confidence bands.

To examine whether and how cultural tightness–looseness, self-control, and self-esteem are associated with the three stigma components, we conducted linear mixed-effects models with covariates (i.e., age, gender, and SES) being controlled. As Figure 4 illustrates, the models revealed that perceived cultural tightness was positively associated with the attribution component (*β* = 0.156, *SE* = 0.079, *t*(251) = 1.990, *p* = 0.048, 95% *CI* [0.002, 0.311]). Self-control was positively associated with the prosocial component (*β* = 0.236, *SE* = 0.095, *t*(251) = 2.491, *p* = 0.013, 95% *CI* [0.049, 0.423]). Self-esteem was positively associated with the exclusionary component (*β* = 0.200, *SE* = 0.065, *t*(251) = 3.050, *p* = 0.003, 95% *CI* [0.071, 0.326]). No other significant associations emerged between cultural or personal factors and the stigma components (*p*s > 0.05). Results were robust in models excluding demographic covariates; the direction and statistical inference for the primary predictors were unchanged (see Supplementary Tables S5–S7 and Figure S1 for detailed results of linear mixed-effects models and robustness tests). Taken together, these findings show that exclusionary, prosocial, and attribution components of stigma show partly distinct association patterns with cultural and personal factors, highlighting their conceptual distinctiveness.

### 3.4. Psychological Network of Disease Stigma

**Figure 5:**
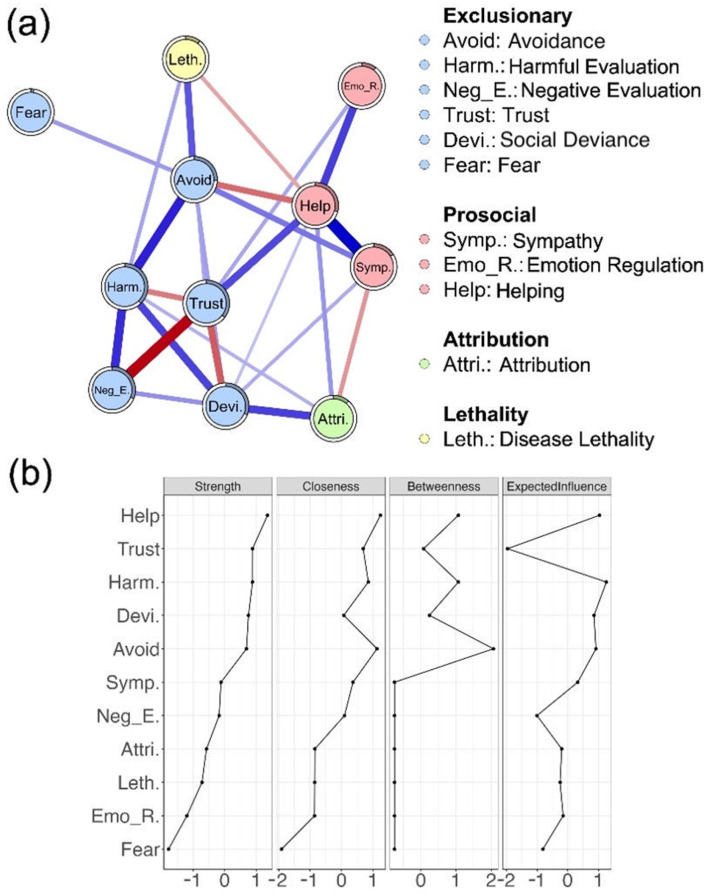
Network analysis. (**a**) Regularized partial correlation network of stigma indicators and disease lethality, colored by stigma component; edge width indicates connection strength. (**b**) Standardized node centrality indices (strength, closeness, betweenness, and expected influence).

As Figure 5a and Table S8 illustrated, the psychological stigma network exhibited a partly modular structure, with the strongest connections emerging within components. Specifically, trust was strongly and negatively connected with negative evaluation within the exclusionary component (edge weight = −0.331, 95% *CI* [−0.370, −0.287]), while sympathy was strongly and positively connected with helping within the prosocial component (edge weight = 0.326, 95% *CI* [0.282, 0.362]). Additional within-component connections and notable cross-component links also emerged. Between exclusionary and prosocial components, a stronger exclusionary node was associated with reduced helping (avoidance—helping, edge weight = −0.145, 95% *CI* [−0.184, −0.104]), alongside modest co-activation with sympathy (avoidance—sympathy, edge weight = 0.127, 95% *CI* [0.084, 0.168]; social deviance—sympathy, edge weight = 0.113, 95% *CI* [0.072, 0.155]). The attribution component bridged the network by aligning with social deviance (edge weight = 0.160, 95% *CI* [0.116, 0.200]) while opposing sympathy (edge weight = −0.119, 95% *CI* [−0.161, −0.073]). These patterns show that exclusionary, prosocial, and attribution components are interconnected rather than isolated, with attribution linking exclusionary and prosocial nodes.

Regarding node centrality (Figure 5b), helping, trust, and harmful evaluation emerged as the most influential nodes, showing the highest strength and expected influence, and relatively elevated closeness and betweenness. In contrast, fear and emotion regulation scored low across all centrality measures, indicating a more peripheral role in the stigma network. Taken together, these results suggest that disease stigma is characterized by a network structure in which certain indicators—helping, trust, and harmful evaluation—exert greater influence, while others, such as fear and emotion regulation, occupy more peripheral positions.

To assess robustness, we conducted 5000-case bootstrapped analyses of edge-weight accuracy and centrality stability. Edge-weight CIs were reasonably narrow (Figure S2). For centrality metrics, correlation stability (CS) coefficients indicated that strength and expected influence were stable under 75% case-drop thresholds (CS = 0.75, Figure S3). Overall, the robustness checks support the reliability of the main structural findings, particularly for strength-based interpretation.

## 4. Discussion

This study identifies three latent psychological components that structure public stigma toward disease-related groups: the exclusionary component capturing avoidance and harmful social responses, the prosocial component reflecting sympathy and helping tendencies, and the attribution component tied to judgments of responsibility. These components emerged consistently across a wide range of disease groups, from infectious conditions (e.g., COVID-19 and Ebola) to non-infectious illnesses (e.g., depression and schizophrenia). These components not only distinguish disease-related stigma from healthy controls but also reveal differences among disease groups. Overall, disease groups exhibited greater exclusionary and prosocial stigma components than healthy controls. Perceived cultural tightness and individual differences (self-control and self-esteem) were differently associated with these components, suggesting that stigma is shaped by a combination of cultural context and personal dispositions. Importantly, given the cross-sectional and self-report nature of the data, these patterns should be interpreted as associations rather than evidence of directional or causal effects. Network analysis further revealed that the three components formed distinct but interconnected clusters, with helping, trust, and harmful evaluation serving as central, highly influential indicators, while other elements, such as fear and emotion regulation, occupied more peripheral positions.

The exclusionary component reflects public tendencies to avoid and socially distance from individuals with illness—responses consistent with behavioral immune system theory (Schaller & Park, 2011). Participants showed heightened exclusionary responses toward groups perceived as more contagious or dangerous (COVID-19, Ebola, and schizophrenia), likely reflecting either infectious threat or behavioral unpredictability—both known triggers for fear-based stigma (Fracchia et al., 1976; Mojtabai, 2010; Oaten et al., 2011). These patterns suggest that exclusionary stigma may be broadly driven by perceived risk, not solely by biological contagion. Individuals with higher self-esteem reported greater exclusion, possibly reflecting identity-protective mechanisms that justify distancing from stigmatized outgroups (Crocker & Major, 1989). The convergence of disease characteristics and self-related motives suggests that exclusionary stigma functions as a defensive social process. However, given the cross-sectional, self-report design, these interpretations remain tentative and cannot adjudicate directionality (e.g., whether self-esteem precedes exclusionary responses or vice versa).

The ambivalence of disease stigma was especially apparent in the prosocial component, which captures compassionate, affiliative responses toward stigmatized groups, including sympathy, emotion regulation, and helping behavior. These reactions may help restore social bonds, particularly when targets are perceived as vulnerable yet non-threatening (Batson et al., 2002; Gärtner et al., 2022). The prosocial dimension in our results—sympathy, helping, and suppression of negative emotions—does not simply index “positive attitudes,” but a mixture of supportive intentions and norm-guided emotion regulation. Against a general “help-the-sick” script, prosocial responding can vary across illness targets when responsibility-implicating appraisals and compassion norms differ. In this light, it is informative that only depression and COVID-19 showed prosocial elevations relative to the healthy control. Both targets are highly salient in everyday life and have been accompanied by prominent public discourse emphasizing support and stigma reduction, which may strengthen injunctive norms for sympathy/helping and for restraining overt negativity (Bagcchi, 2020; Pettigrew & Tropp, 2008). By contrast, HIV/AIDS showed reduced prosocial responding while exhibiting the strongest elevation on the attribution dimension in our data, alongside no difference from the control on the exclusionary dimension. This dissociation is consistent with attribution–affect–action accounts in which stronger responsibility-implicating appraisals dampen pity and helping intentions and reduce motivations to suppress negative affect, even in the absence of heightened overt exclusion (Corrigan et al., 2003; Earnshaw & Chaudoir, 2009; Weiner et al., 1988). Flu may similarly elicit relatively lower prosocial responses when perceived as commonplace and self-manageable. Finally, for targets such as SARS/Ebola and schizophrenia, offsetting mixtures of sympathy and threat-related reactions may yield net differences that are statistically indistinguishable from the control, consistent with ambivalent profiles in stereotype–emotion–behavior frameworks (Cuddy et al., 2007; Fiske, 2002). These findings challenge the notion that stigma uniformly leads to rejection; under some conditions, it may evoke affiliative motives, possibly shaped by social norms of fairness or empathy. Importantly, we also found that individuals with higher self-control reported stronger prosocial responses, suggesting that regulatory capacity may be consistent with the idea that suppression of negative impulses and the enactment of socially valued, supported behaviors (Hofmann et al., 2009; Tangney et al., 2004). Such findings highlight that prosocial reactions may depend not only on contextual appraisals of the stigmatized group but also on personal resources that enable effortful regulation of initial biases. The prosocial component of disease stigma may also function as a subtle expression of power or social conformity (Dovidio et al., 2017a), especially when motivated by reputational concerns. This dynamic raises questions about the authenticity and long-term effectiveness of such responses as strategies for reducing stigma.

Attribution also emerged as a separable component, which captures the tendency to assign blame to individuals for their illness, often based on perceptions of controllability or moral failing (S. Graham, 1997; Weiner, 1995). Consistent with this view, attributional stigma was higher for diseases like HIV/AIDS, which are commonly perceived as more controllable—understood as preventable and behavior-linked—thereby intensifying responsibility and blame attributions and, in turn, shaping downstream emotional and behavioral responses (e.g., reduced pity and support, increased moralized condemnation), as outlined in attribution–affect–action models (McLeod et al., 2025; Švab, 2011). This pattern aligns with neuroimaging evidence showing decreased activation in empathy-related regions (e.g., anterior cingulate cortex) when viewing individuals deemed responsible for their condition, versus increased activation in mentalizing regions when conditions are seen as externally caused (Krendl et al., 2013; Singer et al., 2004). Perceived cultural tightness was positively associated with personal attribution tendencies, suggesting that such judgments may reflect moral identity and uphold normative social order (Crandall & Eshleman, 2003; Gelfand et al., 2011; Hegarty & Golden, 2008). While attribution may appear to be a rational cognitive process, it also legitimizes stigmatizing attitudes and justifies reduced helping behavior, underscoring its ambivalent role in sustaining social exclusion.

The network analysis provides converging evidence for the structural validity of the three stigma components by revealing distinct yet interconnected subclusters. Within the exclusionary component, nodes such as trust, negative evaluation, and avoidance formed tightly coupled relations, with strong negative links between trust and negative evaluation and positive associations between avoidance and harmful evaluation. These patterns underscore the coherence of the exclusionary component, highlighting the close link between negative evaluation and avoidance responses to disease groups, consistent with behavioral immune system accounts (Schaller & Park, 2011). The prosocial component was characterized by strong connections between sympathy and helping, with trust positioned closely. This pattern aligns with the Stereotype Content Model, which predicts that warmth-related appraisals elicit affiliative rather than hostile responses (Fiske, 2002), and with evidence that empathic concern promotes helping (Batson, 2011). By contrast, the attribution component appeared more peripherally located, bridging cognitive judgments with emotional and behavioral responses. Attribution showed negative associations with sympathy and helping, consistent with attribution theory linking blame to reduced prosociality (Weiner, 1995)—and positive associations with deviance-related evaluations, aligning with moralization processes that connect norm violation with culpability (J. Graham et al., 2013), which highlights attribution as a cognitive mechanism that shapes how emotional reactions translate into behavioral responses. Finally, disease lethality displayed a cross-component profile that associated both with threat-driven avoidance and harm and with sympathy and reduced attribution, consistent with the idea that conditions viewed as severe and uncontrollable elicit pity rather than blame (Weiner et al., 1988). Together, these findings indicate that the exclusionary component tracks perceived threat, the prosocial component clusters around warmth and empathy, and the attribution component reflects perceived controllability, supporting the functional distinctiveness of the three components.

Several limitations should be acknowledged. First, the exclusive reliance on a Chinese sample may limit generalizability; cross-cultural studies are needed to test measurement invariance and replicability across diverse contexts (Gelfand et al., 2011). Second, our operationalization of the prosocial/affirmation component relied on a limited set of indicators (e.g., empathy/help-oriented reactions), and future work should incorporate a broader and more validated battery (e.g., standardized empathy, compassion, and altruism measures) to strengthen construct convergence and discriminant validity. Third, the predictors examined here—perceived cultural tightness, self-esteem, and self-control—do not exhaust the ideological and motivational roots of stigma; adding variables such as empathy, social dominance orientation, authoritarianism, or pathogen sensitivity could provide a more comprehensive account. Fourth, we did not assess participants’ stigma-relevant identities or differential exposure to stigma (e.g., sexual orientation) (Flesia et al., 2023), nor did we measure participants’ personal experience with or diagnosis of the focal conditions. These background factors may systematically moderate stigma responses and should be incorporated in future research. Fifth, we did not include other theoretically informative condition categories (e.g., chronic non-communicable illnesses such as diabetes, cancer, or chronic pain/fibromyalgia, or visible vs. invisible conditions). Future work that systematically expands condition types (e.g., chronic non-communicable illnesses, identity-linked stigmas) could test the generalizability of the three-component structure across stigma domains and clarify which appraisal dimensions (e.g., contagion, controllability, norm-violation cues) drive variation in exclusionary, prosocial, and attributional responses. Sixth, the design was cross-sectional and self-report, which precludes causal inference; behavioral or manipulative approaches will be valuable in testing temporal dynamics; all reported effects should be interpreted as associations, and behavioral, longitudinal, or experimental approaches will be valuable in testing temporal dynamics and alternative directions. Finally, the EBIC-glasso solution was relatively dense, suggesting caution in interpreting the smallest edges despite bootstrapped confidence intervals and acceptable centrality stability coefficients (Epskamp et al., 2018).

In conclusion, by integrating dimensional modeling, multilevel regression, and network analysis with stability checks, the current study offers a novel framework for understanding the psychological structure of disease stigma. Rather than treating stigma as a unitary construct, our results highlight its multidimensional nature—comprising the exclusionary, prosocial, and attribution components—each with distinct patterns and potential origins. We offer a multidimensional, theory-anchored stigma framework, which enables more precise measurement, facilitates cross-cultural comparisons, and guides the development of targeted interventions to reduce stigma in diverse social contexts.

## Data Availability

The datasets generated and analyzed during the current study are available in the repository at: https://osf.io/h6kr8/overview?view_only=c4cdb966f4304ff9bc112f3a122dbf9f (accessed on 10th February 2026). All analyses were conducted in R (v4.3.3) and all packages are available at the Comprehensive R Archive Network (CRAN). The main packages included *bootnet* (v1.0), *lavaan* (v0.6-19), *lme4* (v1.1-37), *lmerTest* (v3.1-3), *mgm* (v1.2-15), *FactoMineR* (v2.11), *ggplot2* (v3.5.2), *psych* (v2.5.3), and *emmeans* (v1.11.0). All analysis scripts supporting the findings of the study are openly available in the same OSF project.

## Supplementary Materials

The following supporting information can be downloaded at: https://www.mdpi.com/article/10.3390/bs16020295/s1. Text S1. Disease Targets and Objective Lethality. Figure S1. Linear Mixed Model (LMM) results for each disease group (effects of cultural tightness-looseness, self-control, and self-esteem on baseline-corrected stigma component scores across disease groups). Figure S2. Confidence interval (CI) plots for bootstrapped edge-weight accuracy in the stigma network. Figure S3. Case-drop bootstrapped correlation stability (CS) coefficients for node centrality measures in the stigma network. Table S1. Questionnaire items and factor loadings from the Multiple Factor Analysis. Table S2. Descriptive statistics of stigma component scores by disease. Table S3. Omnibus results of repeated-measures ANOVA for stigma components across disease conditions. Table S4. Pairwise comparisons of estimated marginal means (Bonferroni-corrected) for stigma components across disease conditions. Table S5. Fixed effects from baseline-corrected linear mixed-effects models predicting stigma component scores, with demographic covariates (Δ = disease − healthy). Table S6. Fixed effects from robust baseline-corrected linear mixed-effects models predicting stigma component scores, without demographic covariates (Δ = disease − healthy). Table S7. Likelihood ratio tests comparing full models with demographic covariates versus robustness models without demographic covariates (Δ = disease − healthy). Table S8. Edge weights and 95% bootstrap confidence intervals estimated by EBIC-glasso. References (Chan-Yeung & Xu, 2003), (Chin, 2000), (Garske et al., 2017), (Russell et al., 2020), and (Simonsen et al., 2018) are cited in the Supplementary Materials.
