# Supplementary material for: Psychological Components of Disease Stigma Across Illnesses: Associations with Cultural and Personal Factors
Source: Behav Sci (Basel). 2026 Feb 19;16(2):295. doi: 10.3390/bs16020295 (PMC12937942; doi:10.3390/bs16020295)
Supplement: Supplementary file 1 [file behavsci-16-00295-s001.zip › behavsci-4094970-supplementary.pdf]

Supplementary Materials for

Psychological Components of Disease Stigma Across Illnesses: Associations with Cultural and Personal Factors

This file includes:

S1. Disease Targets and Objective Lethality

S2. Supplementary Figures S1-S3

S3. Supplementary Tables S1-S8

S4. Supplementary References

S1. Disease Targets and Objective Lethality

To ensure broad coverage of disease stigma, we selected seven target conditions to sample key stigma-relevant dimensions, including infectious threat severity, transmission mode, and the inclusion of non-communicable mental health conditions. Specifically, we included three respiratory-transmitted infectious diseases—COVID-19 (CFR = 2.6%) (Russell et al., 2020), Flu (CFR = 0.4%) (Simonsen et al., 2018), and SARS (CFR = 11%) (Chan-Yeung & Xu, 2003)—to represent a within-route gradient of threat severity. We additionally included Ebola (CFR = 62.9%) (Garske et al., 2017) and HIV (CFR of the untreated = 90%) (Chin, 2000) to capture variation in transmission routes (e.g., bodily fluid-transmitted and blood/sexually transmitted diseases) and high perceived severity. To extend applicability of the framework beyond infectious diseases, we included two non-communicable psychological conditions—depression and schizophrenia—which are frequently discussed in the stigma literature. A healthy control target was included as a baseline to account for potential rating tendencies unrelated to disease labels.

S2. Supplementary Figures

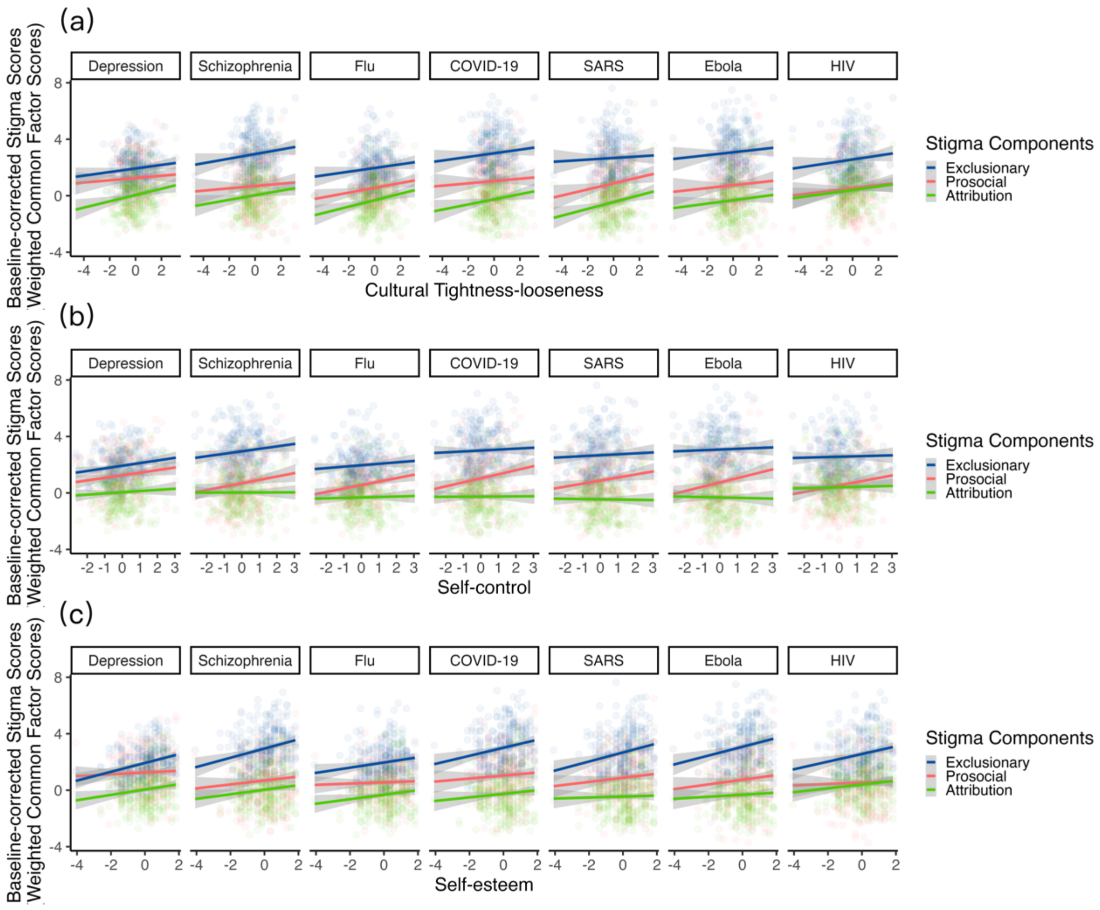

Figure S1. Linear Mixed Model (LMM) results for each disease group. This figure

illustrates the effects of three influencing factors—(a) cultural tightness looseness, (b) self-control, and (c) self-esteem—on the three stigma components (Exclusionary, Prosocial, and Attribution) across different disease groups. Each panel presents baseline-corrected stigma scores (common factors scores) as a function of the corresponding predictor variable for individual disease groups. the regression lines represent the overall trends within each group, capturing the relationship between the influencing factor and stigma components.

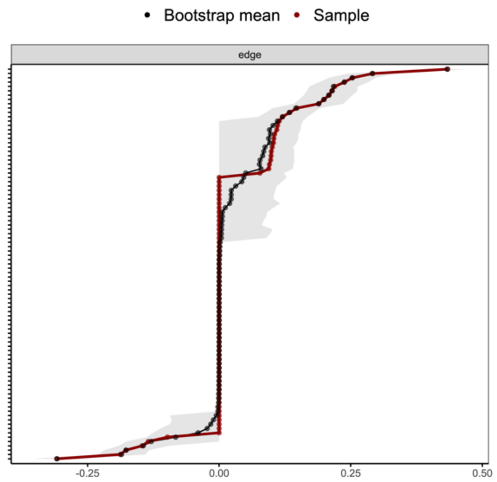

Figure S2. Confidence Interval (CI) plots for bootstrapped edge-weight accuracy in the stigma network.

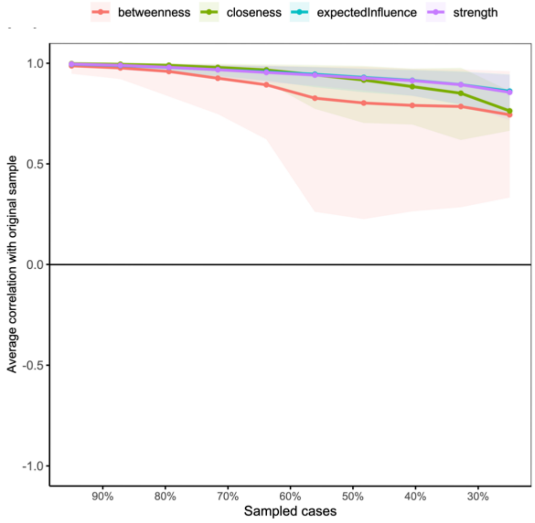

Figure S3. Case-drop bootstrapped Correlation Stability (CS) coefficients for node centrality measures in the stigma network.

S3. Supplementary Tables

Table S1. Questionnaire Items and Factor Loadings from the Multiple Factor Analysis

Item	Psychological Process	Exclusionary Component	Prosocial Component	Attribution Component
I feel fear towards them.	Emotional reactions	0.356	−0.167	−0.007
I feel sympathy towards them.		0.517	0.658	0.045
I suppress my negative emotions.	Cognitive processes	−0.323	0.624	0.042
I attribute their current situation to factors within themselves.		−0.161	−0.210	0.950
I help them.	Withdrawal/approach motivations	−0.361	0.656	0.230
I avoid them.		0.807	0.105	0.011
I think they deviate from social norms.	Social evaluations	0.617	0.134	0.327
I trust them.		−0.635	0.160	−0.118
I think they are harmful to the society.		0.710	0.053	0.214
I think they are generally not a good person.		0.701	0.050	0.140

Table S2. Descriptive statistics of stigma component scores by disease

Component	Group	*Mean*	*SD*	95% CI Lower	95% CI Upper
Exclusionary Component	the Healthy	−1.670	0.713	−1.760	−1.590
	COVID-19	0.542	0.677	0.461	0.623
	SARS	0.298	0.783	0.204	0.392
	Ebola	0.596	0.736	0.508	0.684
	Flu	−0.226	0.717	−0.312	−0.140
	HIV/AIDS	0.217	0.773	0.124	0.309
	Depression	−0.255	0.634	−0.331	−0.179
	Schizophrenia	0.502	0.652	0.424	0.580
Prosocial Component	the Healthy	−0.652	0.992	−0.771	−0.533
	COVID-19	0.295	0.927	0.183	0.406
	SARS	0.147	0.976	0.030	0.264
	Ebola	0.031	1.010	−0.090	0.152
	Flu	−0.142	0.843	−0.243	−0.041
	HIV/AIDS	−0.147	1.03	−0.270	−0.024
	Depression	0.482	0.849	0.380	0.583
	Schizophrenia	−0.013	0.958	−0.128	0.101
Attribution Component	the Healthy	0.084	1.020	−0.039	0.207
	COVID-19	−0.148	0.992	−0.267	−0.029

SARS	−0.339	0.921	−0.450	−0.229
Ebola	−0.201	0.984	−0.319	−0.083
Flu	−0.211	0.913	−0.320	−0.101
HIV/AIDS	0.514	0.935	0.402	0.627
Depression	0.145	0.974	0.028	0.261
Schizophrenia	0.155	0.989	0.037	0.274

Table S3. Omnibus results of repeated-measures ANOVA for stigma components across disease conditions

**Component**	df_1	df_2	F	p	**partial** η^2	**G-G** ε
Exclusionary	4.507	1199.028	454.321	<0.001	0.631	*NA*
Prosocial	3.918	1042.272	57.651	<0.001	0.178	*NA*
Attribution	5.119	1361.577	34.935	<0.001	0.116	*NA*

Note. Greenhouse-Geisser (G-G) epsilon correlations are only reported when sphericity assumptions were violated; otherwise, *NA* indicates no correlation was applied.

Table S4. Pairwise comparisons of estimated marginal means (Bonferroni-corrected) for stigma components across disease conditions

**Component**	**Group (A)**	**Group (B)**	**Mean Differences** (A-B)	*SE*	*p*	**95% CI Lower**	**95% CI Upper**
Exclusionary	Healthy	Covid	−2.215	0.067	<0.001	−2.427	−2.004
	Healthy	SARS	−1.971	0.069	<0.001	−2.187	−1.755
	Healthy	Ebola	−2.269	0.069	<0.001	−2.486	−2.052
	Healthy	Flu	−1.447	0.057	<0.001	−1.627	−1.268
	Healthy	AIDS	−1.890	0.066	<0.001	−2.098	−1.683
	Healthy	Depression	−1.419	0.052	<0.001	−1.583	−1.255
	Healthy	Schizophrenia	−2.175	0.064	<0.001	−2.378	−1.973
	Covid	SARS	0.244	0.036	<0.001	0.131	0.358
	Covid	Ebola	−0.054	0.036	1.000	−0.166	0.059
	Covid	Flu	0.768	0.048	<0.001	0.618	0.919
	Covid	AIDS	0.325	0.043	<0.001	0.190	0.461
	Covid	Depression	0.797	0.046	<0.001	0.651	0.943
	Covid	Schizophrenia	0.040	0.041	1.000	−0.088	0.168
	SARS	Ebola	−0.298	0.036	<0.001	−0.411	−0.185
	SARS	Flu	0.524	0.045	<0.001	0.381	0.667
	SARS	AIDS	0.081	0.045	1.000	−0.061	0.223
	SARS	Depression	0.552	0.047	<0.001	0.405	0.700
	SARS	Schizophrenia	−0.204	0.044	<0.001	−0.344	−0.064
	Ebola	Flu	0.822	0.051	<0.001	0.662	0.982
	Ebola	AIDS	0.379	0.042	<0.001	0.246	0.512
	Ebola	Depression	0.850	0.048	<0.001	0.698	1.002

	Ebola	Schizophrenia	0.094	0.043	0.858	−0.042	0.230
	Flu	AIDS	−0.443	0.049	<0.001	−0.598	−0.288
	Flu	Depression	0.029	0.043	1.000	−0.107	0.164
	Flu	Schizophrenia	−0.728	0.052	<0.001	−0.891	−0.565
	AIDS	Depression	0.471	0.047	<0.001	0.323	0.620
	AIDS	Schizophrenia	−0.285	0.046	<0.001	−0.429	−0.142
	Depression	Schizophrenia	−0.757	0.040	<0.001	−0.884	−0.630
Prosocial	Healthy	Covid	−0.947	0.087	<0.001	−1.222	−0.671
	Healthy	SARS	−0.799	0.085	<0.001	−1.068	−0.531
	Healthy	Ebola	−0.683	0.095	<0.001	−0.983	−0.384
	Healthy	Flu	−0.510	0.066	<0.001	−0.720	−0.301
	Healthy	AIDS	−0.505	0.091	<0.001	−0.793	−0.217
	Healthy	Depression	−1.134	0.076	<0.001	−1.374	−0.893
	Healthy	Schizophrenia	−0.639	0.090	<0.001	−0.921	−0.356
	Covid	SARS	0.147	0.043	0.022	0.011	0.284
	Covid	Ebola	0.263	0.040	<0.001	0.136	0.391
	Covid	Flu	0.437	0.053	<0.001	0.268	0.605
	Covid	AIDS	0.442	0.052	<0.001	0.278	0.605
	Covid	Depression	−0.187	0.056	0.027	−0.364	−0.010
	Covid	Schizophrenia	0.308	0.053	<0.001	0.142	0.474
	SARS	Ebola	0.116	0.043	0.196	−0.019	0.251
	SARS	Flu	0.289	0.055	<0.001	0.116	0.462
	SARS	AIDS	0.294	0.054	<0.001	0.125	0.464
	SARS	Depression	−0.334	0.057	<0.001	−0.514	−0.155
	SARS	Schizophrenia	0.161	0.055	0.100	−0.012	0.333
	Ebola	Flu	0.173	0.059	0.097	−0.012	0.358
	Ebola	AIDS	0.178	0.049	0.011	0.022	0.334
	Ebola	Depression	−0.451	0.062	<0.001	−0.646	−0.255
	Ebola	Schizophrenia	0.045	0.053	1.000	−0.124	0.213
	Flu	AIDS	0.005	0.061	1.000	−0.188	0.198
	Flu	Depression	−0.624	0.054	<0.001	−0.795	−0.452
	Flu	Schizophrenia	−0.129	0.058	0.783	−0.312	0.055
	AIDS	Depression	−0.629	0.062	<0.001	−0.824	−0.433
	AIDS	Schizophrenia	−0.134	0.056	0.486	−0.309	0.042
	Depression	Schizophrenia	0.495	0.057	<0.001	0.316	0.674
Attribution	Healthy	Covid	0.232	0.087	0.232	−0.043	0.507
	Healthy	SARS	0.423	0.083	<0.001	0.162	0.684
	Healthy	Ebola	0.285	0.089	0.045	0.003	0.567
	Healthy	Flu	0.294	0.078	0.005	0.048	0.540
	Healthy	AIDS	−0.431	0.082	<0.001	−0.688	−0.173
	Healthy	Depression	−0.061	0.080	1.000	−0.312	0.190
	Healthy	Schizophrenia	−0.072	0.081	1.000	−0.327	0.184
	Covid	SARS	0.191	0.049	0.003	0.037	0.346
	Covid	Ebola	0.053	0.053	1.000	−0.114	0.220

Covid	Flu	0.063	0.054	1.000	−0.109	0.234
Covid	AIDS	−0.662	0.066	<0.001	−0.871	−0.454
Covid	Depression	−0.293	0.063	<0.001	−0.492	−0.093
Covid	Schizophrenia	−0.303	0.062	<0.001	−0.499	−0.107
SARS	Ebola	−0.138	0.051	0.206	−0.300	0.023
SARS	Flu	−0.129	0.049	0.273	−0.285	0.027
SARS	AIDS	−0.854	0.064	<0.001	−1.054	−0.653
SARS	Depression	−0.484	0.064	<0.001	−0.687	−0.280
SARS	Schizophrenia	−0.495	0.063	<0.001	−0.694	−0.295
Ebola	Flu	0.010	0.055	1.000	−0.163	0.182
Ebola	AIDS	−0.715	0.064	<0.001	−0.916	−0.515
Ebola	Depression	−0.345	0.069	<0.001	−0.563	−0.128
Ebola	Schizophrenia	−0.356	0.065	<0.001	−0.561	−0.152
Flu	AIDS	−0.725	0.062	<0.001	−0.921	−0.529
Flu	Depression	−0.355	0.062	<0.001	−0.551	−0.160
Flu	Schizophrenia	−0.366	0.058	<0.001	−0.550	−0.182
AIDS	Depression	0.370	0.067	<0.001	0.159	0.581
AIDS	Schizophrenia	0.359	0.060	<0.001	0.168	0.550
Depression	Schizophrenia	−0.011	0.049	1.000	−0.166	0.144

Table S5. Fixed effects from baseline-corrected linear mixed-effects models predicting stigma component scores, with demographic covariates (Δ = disease – healthy)

Component	Predictor	*β*	*SE*	*t*	*p*	95% CI Lower	95% CI Upper
Exclusionary	Cultural Tightness-looseness	0.085	0.053	1.600	0.111	−0.022	0.213
	Self-Control	−0.048	0.067	−0.712	0.477	−0.179	0.084
	Self-Esteem	0.200	0.065	3.050	0.002	0.071	0.328
Prosocial	Cultural Tightness-looseness	0.024	0.085	0.288	0.773	−0.143	0.191
	Self-Control	0.236	0.095	2.491	0.013	0.049	0.423
	Self-Esteem	−0.009	0.093	−0.098	0.922	−0.192	0.174
Attribution	Cultural Tightness-looseness	0.156	0.079	1.990	0.048	0.002	0.311
	Self-Control	−0.143	0.088	−1.625	0.105	−0.317	0.030
	Self-Esteem	0.104	0.086	1.202	0.230	−0.066	0.273

Note. Outcomes are baseline-corrected component scores (Δ = disease – Healthy). Predictors (cultural tightness-looseness, self-control, self-esteem, age) were standardized. Education, SES, gender, and disease target were included as covariates but coefficients are omitted for brevity (available in shared code/data).

Table S6. Fixed effects from robust baseline-corrected linear mixed-effects models predicting stigma component scores, without demographic covariates (Δ = disease – healthy)

Component	Predictor	*β*	*SE*	*t*	*p*	95% CI Lower	95% CI Upper
Exclusionary	Cultural Tightness-looseness	0.039	0.053	0.736	0.462	-0.065	0.143
	Self-Control	-0.048	0.059	0.736	0.417	-0.163	0.067

	Self-Esteem	0.206	0.058	3.531	0.0005	0.092	0.321
Prosocial	Cultural Tightness-looseness	0.042	0.057	0.731	0.465	-0.070	0.153
	Self-Control	0.156	0.063	2.471	0.014	0.032	0.271
	Self-Esteem	-0.018	0.063	-0.294	0.769	-0.141	0.104
Attribution	Cultural Tightness-looseness	0.124	0.055	2.259	0.025	0.016	0.231
	Self-Control	-0.062	0.061	-1.028	0.305	-0.181	0.056
	Self-Esteem	0.092	0.060	1.523	0.129	-0.026	0.210

Note. Outcomes are baseline-corrected component scores (Δ = disease – Healthy). Predictors (cultural tightness-looseness, self-control, self-esteem, age) were standardized. To compare model performance, no demographic variables were included as covariates.

Table S7. Likelihood ratio tests comparing full models with demographic covariates versus robustness models without demographic covariates (Δ = disease – healthy)

**Component**	**Model**	***AIC***	***BIC***	***Log-Likelihood***	χ^2	***df***	***p***
Exclusionary	Without demographic	3352.1	3390.8	-1669.0			
	Full model	3355.0	3460.1	-1658.5	21.102	12	0.049
Prosocial	Without demographic	3209.3	3248	-1597.6			
	Full model	3325.8	3331	-1593.9	7.441	12	0.827
Attribution	Without demographic	3359.8	3598.6	-1772.9			
	Full model	3567.0	3672.2	-1764.5	16.79	12	0.158

Note. Each likelihood ratio test (LRT) compares a full model including demographic covariates (age, gender, education, SES; fixed effects) with a reduced model excluding these covariates, while retaining the same primary predictors and random intercept structure. Models were fit using maximum likelihood. The table reports results from the LRT. A non-significant LRT indicates that adding demographic covariates did not significantly improve model fit.

Table S8. Edge weights and 95% bootstrap confidence intervals estimated by EBIC-glasso.

**Node (A)**	**Node (B)**	**Estimate**	**95% CI Lower**	**95% CI Upper**
Negative Evaluation	Trust	−0.331	−0.370	−0.287
Helping	Sympathy	0.326	0.282	0.362
Avoidance	Disease Lethality	0.325	0.285	0.363
Harmful Evaluation	Negative Evaluation	0.243	0.200	0.289
Avoidance	Harmful Evaluation	0.235	0.190	0.278
Deviance	Harmful Evaluation	0.223	0.178	0.267
Emotion Regulation	Helping	0.208	0.164	0.252
Helping	Trust	0.200	0.157	0.241
Deviance	Trust	−0.198	−0.240	−0.153
Attribution	Disease Lethality	−0.180	−0.217	−0.139
Attribution	Deviance	0.160	0.116	0.200
Disease Lethality	Sympathy	0.151	0.111	0.192
Deviance	Negative Evaluation	0.150	0.107	0.194

Avoidance	Helping	−0.145	−0.184	−0.104
Harmful Evaluation	Trust	−0.141	−0.186	−0.095
Avoidance	Sympathy	0.127	0.084	0.168
Attribution	Sympathy	−0.119	−0.161	−0.073
Harmful Evaluation	Disease Lethality	0.116	0.074	0.160
Avoidance	Deviance	0.114	0.069	0.157
Deviance	Sympathy	0.113	0.072	0.155

Note. Estimate: edge weight (partial correlation) estimated by EBIC-glasso. 95% CI: nonparametric bootstrap confidence intervals. Out of 45 possible edges among 10 nodes, 29 edges were estimated by EBIC-glasso. Of these, 20 edges (68.97%) were significant.

S4. Supplementary References

Chan-Yeung, M., & Xu, R. H. (2003). SARS: epidemiology. *Respirology*, *8*, S9-14. https://doi.org/10.1046/j.1440-1843.2003.00518.x

Chin, J. (2000). Control of communicable diseases manual. American Public Health Association.

Garske, T., Cori, A., Ariyarajah, A., Blake, I. M., Dorigatti, I., Eckmanns, T., Fraser, C., Hinsley, W., Jombart, T., Mills, H. L., Nedjati-Gilani, G., Newton, E., Nouvellet, P., Perkins, D., Riley, S., Schumacher, D., Shah, A., Van Kerkhove, M. D., Dye, C., . . . Donnelly, C. A. (2017). Heterogeneities in the case fatality ratio in the West African Ebola outbreak 2013-2016. *Philosophical Transactions of the Royal Society B: Biological Sciences*, *372*(1721), 20160308. https://doi.org/10.1098/rstb.2016.0308

Russell, T. W., Hellewell, J., Jarvis, C. I., van Zandvoort, K., Abbott, S., Ratnayake, R., group, C. C.-w., Flasche, S., Eggo, R. M., Edmunds, W. J., & Kucharski, A. J. (2020). Estimating the infection and case fatality ratio for coronavirus disease (COVID-19) using age-adjusted data from the outbreak on the Diamond Princess cruise ship, February 2020. *Eurosurveillance*, *25*(12), 2000256. https://doi.org/10.2807/1560-7917.ES.2020.25.12.2000256

Simonsen, L., Higgs, E., Taylor, R. J., Wentworth, D., Cozzi-Lepri, A., Pett, S., Dwyer, D. E., Davey, R., Lynfield, R., Losso, M., Morales, K., Glesby, M. J., Weckx, J., Carey, D., Lane, C., Lundgren, J., Insight, F. L. U., & Groups, F. L. U. S. (2018). Using Clinical Research Networks to Assess Severity of an Emerging Influenza Pandemic. *Clinical Infectious Diseases*, *67*(3), 341-349. https://doi.org/10.1093/cid/ciy088
